# Input-Independent Energy Harvesting in Bistable Lattices from Transition Waves

**DOI:** 10.1038/s41598-018-22003-7

**Published:** 2018-02-26

**Authors:** Myungwon Hwang, Andres F. Arrieta

**Affiliations:** School of Mechanical Engineering, Purdue University, West Lafayette, Indiana 47907 USA

## Abstract

We demonstrate the utilisation of transition waves for realising input-invariant, frequency-independent energy harvesting in 1D lattices of bistable elements. We propose a metamaterial-inspired design with an integrated electromechanical transduction mechanism to the unit cell, rendering the power conversion capability an intrinsic property of the lattice. Moreover, focusing of transmitted energy to desired locations is demonstrated numerically and experimentally by introducing engineered defects in the form of perturbation in mass or inter-element forcing. We achieve further localisation of energy and numerically observe a breather-like mode for the first time in this type of lattice, improving the harvesting performance by an order of magnitude. Our approach considers generic bistable unit cells and thus provides a universal mechanism to harvest energy and realise metamaterials effectively behaving as a capacitor and power delivery system.

## Introduction

The utilisation of nonlinearity in vibration-based energy harvesting has been widely studied for improving the restricted bandwidth in which energy can be efficiently converted^1–13^. Recently, the high tunability of the band structures in phononic crystals has drawn a significant attention from researchers as an alternate route to achieve the broad-band energy harvesting^14–16^. However, the fundamental reliance on mechanical resonances and band gaps to convert energy from oscillatory sources restricts its applicability to finite frequency bands. This problem is exacerbated by the dimensional limit constraining resonant conversion at low structural frequencies in view of the inverse relationship between size and natural frequency; harvesting at low frequencies implies the use of impractically large devices. The research efforts have been extended to metamaterial-based energy harvesting concepts^17–22^, whose locally resonant feature offers unit cell designs in subwavelength scales and more tuning flexibility in band gap formation^23–25^; several authors have specifically addressed the challenge of the scalability^26,27^. Nonetheless, the optimal operating frequencies are still relatively high compared to the typical frequency range of the structural sources^28,29^, and their harvesting performances depend on the frequency, types, and strengths of the input excitations. Moreover, the operation principle through focusing dispersive waves fundamentally constrains the robust harvesting of energy to a small specific area only.

Nonlinear metamaterials capable of sustaining solitary waves for a broad class of inputs may simultaneously address the problems posed by dispersion, high frequency, and narrow-band conversion. In the context of a granular chain^30^, ref.^31^ has demonstrated the harvesting of energy from solitary waves arising from low-frequency impulse excitations using a nonlinear medium itself as a means to concentrate energy to a focal point, where piezoelectric conversion is conducted. Ref.^32^ has laid a foundational work on harvesting energy from non-topological solitary waves within a granular lattice through an embedded piezoelectric sensor. However, the study of topological solitons and associated dynamics for energy harvesting has received much less attention.

**Figure 1:**
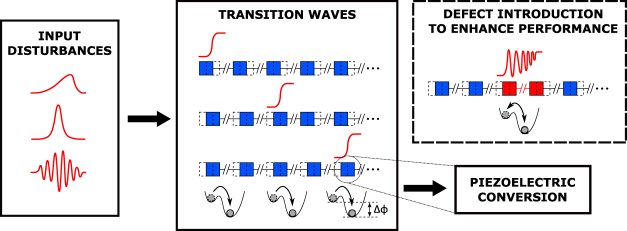
Conceptual work flow of energy harvesting from transition waves in bistable lattices.

We demonstrate that the invariance of transition waves to different boundary inputs in 1D lattices of bistable elements^33–35^ enables the concentration, transmission, and harvesting of input energy independently from the excitations. We calculate the amount of available energy to be harvested from the topological transition waves and introduce electromechanical unit cells, allowing for the design of metamaterials with inherent capacity to harvest energy carried in the form of transition waves. In addition, engineered defects^36–41^ are utilised to further manipulate the lattice dynamics. A single or distributed defect(s) introducing mass and/or inter-element forcing perturbations to the lattice periodicity allow us to focus energy in a specific lattice region, enabling an efficient method for transduction. Our numerical simulations reveal the presence of breather-like modes, enabling the transduction of energy triggered by a transition wave into an oscillatory form, thereby creating a mechanism to deliver power from the lattice over a longer time period. This system behaves as a mechanical capacitor with a tunable discharge rate capable of delivering electrical energy. The used bistable unit cells are generic and can be realised through a broad range of architectures^42–45^. Therefore, the uncovered dynamics allow for creating a general robust lattice-intrinsic mechanism for transmitting, focusing, and converting energy from motion, giving rise to inherently harvesting metamaterials as illustrated in Fig. 1.

## Results and Discussion

### Bistable Lattice Model

**Figure 2:**
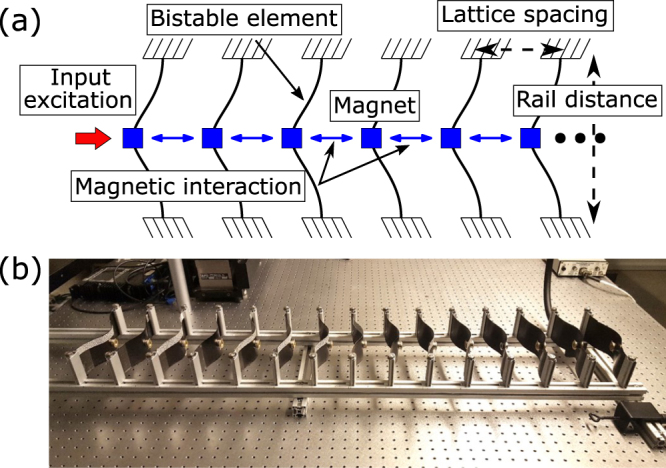
(**a**) Schematics and (**b**) experimental setup of the lattice with bi-stable unit cells.

The studied model is based on a 1D periodic lattice of bistable elements connected by inter-element magnetic forces^34^. Figure 2 shows a schematic representation of the lattice and the experimental setup used throughout. The experimental lattice model is built using 15 identically prepared bistable elements fabricated from carbon fiber reinforced composite laminates^46^. The input excitation is applied at the first site, and three measuring sites (9^th^, 10^th^, 11^th^) within the lattice are selected for the analyses to minimise any unwanted boundary effects. The dynamic response of the lattice is captured by a commercial high-speed digital image correlation system (VIC-3D).

The system is studied numerically with a discrete analytical model governed by the advance-delay differential equation given by

1 $$m{\ddot{u}}_{n}={ {\mathcal F} }_{mag}({u}_{n-1},{u}_{n},{u}_{n+1})+{ {\mathcal F} }_{onsite}({u}_{n})-b{\dot{u}}_{n},$$

where *u*_*n*_ is displacement at *n*^th^ site from the static equilibrium with the higher energy potential, and overdot represents derivative with respect to time. The inter-element magnetic force and the on-site force are given by

2 $$\begin{array}{rcl}{ {\mathcal F} }_{mag} & = & -A{({u}_{n+1}-{u}_{n}+L)}^{p}+A{({u}_{n}-{u}_{n-1}+L)}^{p},\\ { {\mathcal F} }_{onsite} & = & -2{C}_{2}{u}_{n}-3{C}_{3}{u}_{n}^{2}-4{C}_{4}{u}_{n}^{3}.\end{array}$$

The following baseline lattice parameters are used unless specified otherwise: mass *m* = 29.4 g, lattice spacing *L* = 0.07 m, rail distance *R* = 0.225 m, and element damping *b* = 0.52 Ns/m. The choice of element damping is made to approximate the decay rate exhibited by the experimental system. The measured values for the magnetic interaction parameters *A* and *p* and for the coefficients *C*_2_, *C*_3_, and *C*_4_ of the cubic force functions are specified in Supplementary Fig. 1. Newmark-*β* method^47^ is used to numerically study the responses of the unit cells as transition waves propagate. We choose a sufficiently large number of elements (N = 500) to simulate a semi-infinite boundary.

### Response Invariance

In the linear regime, the dispersion relation between the nondimensionalised wave number $\bar{k}$ and frequency $\bar{\omega }$ is obtained as

3 $$\bar{k}=\pm 2\,\arcsin \,\sqrt{\frac{-{\bar{\omega }}^{2}-i\bar{\omega }\bar{b}-{\bar{ {\mathcal F} }^{\prime} }_{onsite}({\bar{u}}_{n}^{\ast })}{4p}}.$$

The presence of an imaginary part contributes to wave attenuation, and thus phonons disintegrate eventually in a physical setup, where damping is always present ($\bar{b}$ > 0) – see Supplementary Information for the derivation of the dispersion relation and phonon transmission.

**Figure 3:**
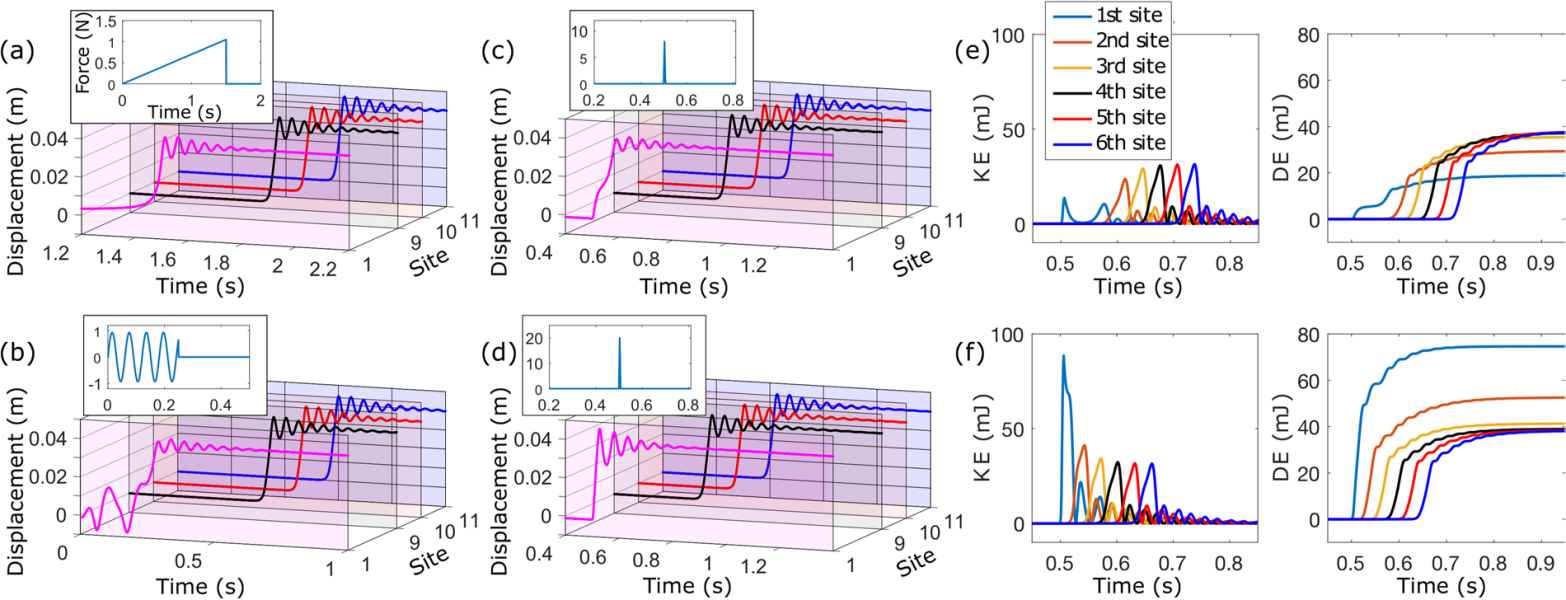
Lattice responses generating transition waves for different input forces applied at 1^st^ site: (**a**) quasi-static, (**b**) sinusoidal within the pass band, and (**c**,**d**) impulse-like loads with different intensities. Kinetic and dissipated energies at each site under impulse-like loads of (**e**) low and (**f**) high levels.

However, a very different aspect can be observed when a transition wave is generated. The time responses of the elements at the input and measuring sites under a quasi-static application of load, sinusoidal excitation, and impulse-like excitation are shown in Fig. 3(a–c), respectively. Additionally, the response under a higher-intensity impulse [Fig. 3(d)] is presented. Although the response form and the time it takes to trigger snapthrough of the first element are different for each excitation condition, the responses at the measuring sites (9^th^, 10^th^, 11^th^) are essentially the same for all four cases. This shows that the waveform is preserved along the path of propagation (except near the lattice boundaries) as long as the initial state transition is triggered by any means, thus input-invariant response.

The invariant lattice response is validated experimentally by using quasi-static and impulsive inputs of various rates and intensities on the demonstrator shown in Fig. 2(b). For all the explored cases, a stable transition wave is formed as long as the first element snaps through to its second state, three of which are shown in Supplementary Videos 1–3. Given the unidirectionality of the lattice theoretically and experimentally shown in ref.^34^, any input triggering a snap-through of any unit cell in the lattice results in the stable propagation of a transition wave. This assures the input-invariant behaviour of the response in the lattice.

Taking advantage of the response invariance at every site, each element forming the lattice can contribute to the harvesting of energy, provided that transduction mechanism is installed at every site. The minimum static force required to initiate the state transition is 0.56 N for a single bistable element (from the force-deflection curve); this value increases to 0.95 N for an array with more than three elements (see Supplementary Fig. 3). Although nearly double amount of input force (thus energy) is needed to trigger the snap-through for the array, the latter snapping force remains unchanged regardless of the size of the array, implying that it can attain a significantly higher energy output to input ratio than a single-element energy harvester does.

### Harvested Energy

To calculate the total energy in the system, the governing equation is multiplied by the unit-cell velocity ${\dot{u}}_{n}$, integrated over time and summed over all elements, yielding

4 $$\sum _{n=1}^{N}\,[\mathop{\underbrace{\frac{1}{2}m{\dot{u}}_{n}^{2}}}\limits_{{\rm{KE}}}+\varphi ({u}_{n})]-\sum _{n=1}^{N-1}\,\frac{A}{p+1}{({u}_{n+1}-{u}_{n}+L)}^{p+1}=-\sum _{n=1}^{N}\,\mathop{\underbrace{\int {b}_{tot}{\dot{u}}_{n}^{2}dt}}\limits_{{\rm{DE}}},$$

where *b*_*tot*_ is the total effective damping, and KE, *ϕ*(*u*_*n*_), and DE are the kinetic, on-site potential, and dissipation energies for each unit cell, respectively. The available energy from the motion of each unit cell may be divided into three parts: (1) the initial vibration from the phonon transmission, (2) the state transition from the high to low energy state, and (3) the oscillatory tail behind the main transition wave front. The first contribution is negligible according to the earlier discussion. The third contribution can be considered as excess energy, which does not affect the stability of the transition wave, and can be advantageously tuned to induce large oscillatory tails for optimizing the harvested energy of the lattice.

Figure 3(e,f) compare the kinetic and dissipated energies at each site under two different levels of impulse excitations. With the interaction between the stored energy (due to the asymmetry in the strain potential) and the damping of the on-site element, any deficiency or excess in input energy is self-adjusted to the level dictated by the lattice’s intrinsic properties, acting as a power regulator. With a marginal input to trigger the snap-though of the first element, the stored potential continues to supply energy over the course of propagation until the state transition is eventually stabilised [Fig. 3(e)]; on the contrary, any excess input energy is rapidly dissipated in the first few elements until the transition motion stabilises [Fig. 3(f)]. For the given baseline experimental lattice setup, the stabilisation occurs in approximately four elements.

Rather than developing a specific coupling relation for each different transduction mechanism, the mechanical to electrical conversion may be represented as an additional damping *d* for simplicity^48^. This gives the total effective damping *b*_*tot*_ = b^*^ + *d*, where b^*^ captures the modified mechanical damping due to the added mechanism. The total harvested energy from a single element can then be calculated as ${\int }_{0}^{\infty }\,d{({\dot{u}}_{n})}^{2}dt$. This is the cumulative energy that the harvester can draw from the motion of a single element. The power output, which is a more standard performance measure in harvesters under cyclic excitations, may also be obtained in the average sense by dividing the cumulative energy by the time period of the input.

**Figure 4:**
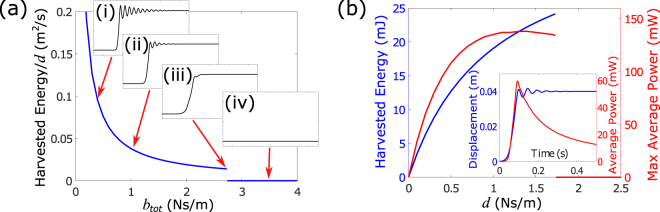
(**a**) Harvested energy factor at a single site of periodic lattice vs. the total damping of the lattice: corresponding responses to values (1) *b*_*tot*_ = 0.4 Ns/m, (2) *b*_*tot*_ = 1.0 Ns/m, (3) *b*_*tot*_ = 2.73 Ns/m, and (4) *b*_*tot*_ = 3.5 Ns/m are plotted in the insets. (**b**) Total harvested energy and maximum average power for a fixed b^*^ = 1.0 Ns/m.

The stability of the transition wave must be ensured in order to allow for the input energy to be transmitted and converted. To study the critical value of the total damping still enabling electromechanical conversion from the transition wave, we define the harvested energy factor as the converted energy from a single element divided by *d* and plot this value as a function of the total damping *b*_*tot*_ in Fig. 4(a). This shows that the transition waves disintegrate for *b*_*tot*_ >2.73 Ns/m, resulting in no harvested energy. For the limiting case *b*_*tot*_ = 2.73 Ns/m, the response shows state change occurring without any oscillatory tails [inset of Fig. 4(a)]. For a given transduction coupling *d*, any reduction in structural damping results in a higher response speed and generation of the greater oscillatory tail, both of which can increase the energy harvested by the system. Assuming b^*^ is fixed, on the other hand, the cumulative harvested energy from a single element monotonically increases with an increasing *d* [blue curve in Fig. 4(b)]. The maximum average power occurs toward the end of the transition motion [inset of Fig. 4(b)]. As a result, there is a trade-off in the maximum average power that can be achieved with increasing *d* since such change tends to slow the transition speed. This behaviour is observed in the maximum average power plot (red curve), where the peak occurs slightly below the limiting value of *d* that allows stable propagation of a transition wave. In the limit, the maximum possible energy that can be converted is the strain energy stored in the lattice, implying the lattice behaves as a mechanical capacitor from which packets of energy can be drawn and transmitted as transition waves.

### Engineered Defects for Energy Harvesting

**Figure 5:**
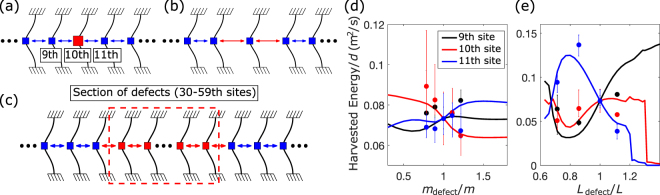
Schematic representations of (**a**) mass defect, (**b**) inter-element forcing defect applied by adjusting the local lattice spacing distance, and (**c**) lattice containing a section of engineered defects. (**d**,**e**) Available energy factor for harvesting under the presence of mass and lattice spacing defects, respectively. The dots represent the experimentally obtained results, and the defect is located at the 10^th^ site.

To best exploit the energy stored within the lattice, engineered defects can be considered as means to concentrate the conversion to a few selected elements, thereby enabling harvesting at specific sites of interest. Defects can thus serve the purpose of a graded layer or region to which energy in the lattice can be directed for efficient conversion using the transition wave as a carrier. Three types of defects are considered: single mass [Fig. 5(a)] and inter-element forcing [Fig. 5(b)] defects, both of which are applied at 10^th^ site in this study, and a region of distributed defects [Fig. 5(c)], which are applied between 30^th^ and 59^th^ sites. Experimentally, mass defects are created by attaching or removing additional nonmagnetic metal parts on the defect elements [red mass in Fig. 5(a)], and inter-element forcing defects are generated by changing the initial lattice spacing distances around the defect [red arrows in Fig. 5(b)].

Figure 5(d,e) show the numerical results of the harvested energy factor as a function of the ratio between the defect and the periodic lattice value for the mass (*m*_*defect*_/*m*) or lattice spacing distance (*L*_*defect*_/*L*): the sharp drop toward the large spacing distance in Fig. 5(e) represents disintegration of the stable propagation of the transition wave. For the studied case, we observe that the lighter the mass defect, the more energy can be harvested from the defect location as less kinetic energy is required to propagate the wave through the site. Two noticeable peaks are observed for the inter-element spacing defect. The harvested energy of the element preceding the defect (9^th^ element) exhibits a non-monotonic behaviour, showing a dip for distances below the periodic baseline with a minimum at *L*_*defect*_/*L* ≈ 0.77 and subsequently reaching the maximum at the limiting spacing value for the stable wave propagation (*L*_*defect*_/*L* ≈ 1.2). On the other hand, the harvested energy available at the element after the defect (11^th^ element) features a peak value at about *L*_*defect*_/*L* ≈ 0.77, then monotonically decreases with the spacing distance. The harvested energy factors calculated from the experimental data after normalisation (see Supplementary Information) are overlaid with error bars, showing the reasonable qualitative match with those attained from the numerical calculation.

The delivery of power over longer periods of time may be created if a larger oscillatory tail is induced. This is achieved via the introduction of a distributed defect region spanning several sites. The amplitude of the tail becomes larger with increasing mass (see Supplementary Information) or decreasing damping [Fig. 4(a)]. In addition, constructive interference occurs from the reflected wave at the boundary from heavy to light or from stiff to soft (equivalent to large to small inter-element forcing) region^49^, which can further increases the amplitude at the boundary.

**Figure 6:**
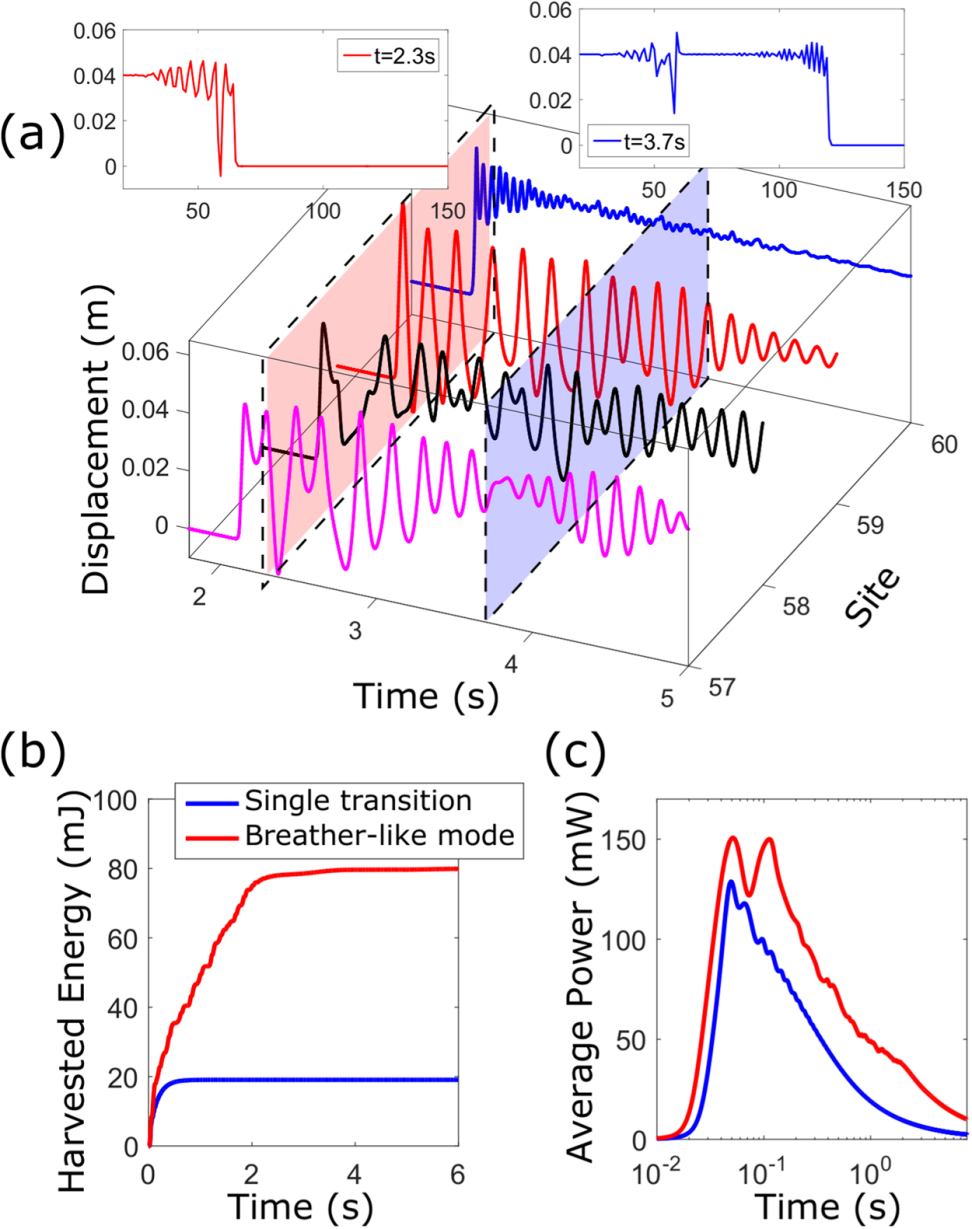
(**a**) Time responses of individual elements near the boundary of the defect section. In the insets are the spatial motions at *t* = 2.3 s and *t* = 3.7 s. (**b**,**c**) Comparison of the harvested energy and average power to the periodic lattice without defects.

Figure 6(a) shows the response when *m*_*defect*_ = 8*m*, *L*_*defect*_ = 0.8*L* and *b*_*tot*_ = 0.2 Ns/m are used, and the defect section is selected to be long enough [30–59^th^ sites as in Fig. 5(c)] to stabilise its response to the lattice-intrinsic level with the modified parameters. Near the boundary of the defect section, the local reflections generate kinks traveling in opposite directions while the global unidirectionality is preserved due to the asymmetry in the strain potential. The interplay between the reflection and the natural tendency of the lattice to travel forward produces spatially localised nonlinear oscillations with several state transitions and large amplitudes, which are the characteristics of a meta-stable breather-like mode^50^ (see Supplementary Video 4 and Fig. 5).

In this example, the harvested energy at the boundary of the defect section (59^th^ site) is calculated to be 80 mJ, compared to 19 mJ in the periodic counterpart, assuming *d* = 0.1 Ns/m is used [Fig. 6(b)]. Recalling the input independence property of the lattice, we harvest four times more energy under the same input energy by virtue of wave localisation in the breather-like mode. The average power output shows no significant gain since its maximum value is mainly governed by the initial state transition [Fig. 6(c)]. However, the persistent localised transitions allow the conversion of significant power over a longer period of time in comparison with a single transition wave. For example, the second peak (150 mW at 0.113 s) occurs at about twice the period of the first peak (151 mW at 0.051 s), while the periodic counterpart experiences a significant power drop from 129 mW to 93 mW during the same time period. Therefore, substantial power output-to-input ratio improvement under cyclic excitation is achieved, rendering robust harvesting possibility.

### Experimental Implementation

The lattice characteristics for energy harvesting are demonstrated using piezoelectric transducers, bonded to the bistable element at the 10^th^ site. The voltage responses are obtained by using the simplest resistive circuit. Following ref.^51^, the cumulative energy and the average power are used as performance measures due to the transient nature of the unit cell dynamic response: the cumulative energy is calculated from the time integral of the power *V* ^2^/*R* over the entire time span, and the average power is obtained by dividing the cumulative energy by the time measured from the onset of the motion.

**Figure 7:**
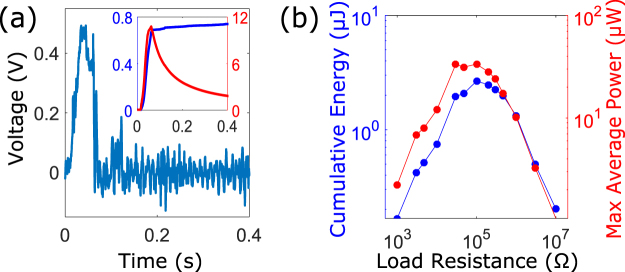
(**a**) Voltage response with 10 *k*Ω resistor and the associated cumulative energy (in blue) and average power (in red) in the inset. (**b**) Resistance sweep of the maximum cumulative energy (in blue) and maximum average power (in red).

Figure 7(a) shows the voltage response of the transduction unit cell for a resistance value of 10 *k*Ω. The attachment of a piezoelectric transducer substantially increases the exhibited damping from *b* to b^*^ + *d*. As a result, the induced vibratory motion on the unit cell is highly damped and most of the harvested energy arises from the state transition. The inset shows the cumulative energy and the average power harvested at the transduction unit cell as a transition wave propagates through the cell. Even at this distant location from the boundary, substantial energy can be harvested. These results are improved further as revealed from the resistor sweep shown in Fig. 7(b). For the baseline values of the experimental lattice, both the cumulative energy and the maximum average power harvested measures are maximised for a value of 100 *k*Ω, reaching peak values of 2.8 *μ*J and 32 *μ*W, respectively.

## Conclusion

We demonstrate that the nonlinear dynamics of a 1D lattice of bistable elements can be utilised to inherently generate invariant energy harvesting capabilities from transition waves. The stored energy from the topological strain difference allows bistable lattices to act as mechanical capacitors, where the fed energy can be harvested during the release process in the form of wave energy transmission. The lattice dynamics are further manipulated through defect inclusions resulting in the emergence of breather-like modes, revealing a mechanism for delivering the stored energy over extended time periods. The input-independent dynamics, extreme directionality and topological energy harvesting characteristics of the presented system allow for producing protective metastructures that concurrently redirect and harvest surplus energy when subject to disruptive events, such as blasts, Tsunamis, or earthquakes. The proposed metastructures thus provide an ideal implementation for protecting critical systems and infrastructure, while providing an intrinsic source for local power to maintain continued operation after natural or man-made disasters.

## Materials and Methods

### Lattice Preparation

Each unit cell comprised a bistable element with a set of grade N42 neodymium magnets (Supermagnete R-19-09-06-N) attached at the center. The unit cells were arranged along the rail on an optical table in a way to exert repulsive forces between themselves, forming a lattice under uniform inter-element forces. The same force-deflection relations from the previous study^34^ were used for the on-site element and the magnet (Supplementary Fig. 1); the data points were obtained in a series of quasi-static compression tests and fitted in a polynomial form and a exponential form, respectively for the bistable element and the magnet. A more detailed measurement procedure can be found in ref.^34^ and the references therein.

### Experimental Data Acquisition

A random black and white speckled pattern was printed on a thin sticky paper and attached on the top edge of each of the bistable elements (Supplementary Fig. 6). The input excitation was triggered through repulsive magnetic force applied by pulling and releasing the elastic translating mount at the first element, and the resulting motions were captured by a set of two high-speed cameras (Photron UX100) with DSLR lenses (NIKKOR 24–85 mm f/2.8-4D IF) at 1000 fps sampling rate. A commercial digital image correlation software (VIC-3D) was used for post-processing, and the displacement of each element was measured at the point closest to the center of the element (bottom of the speckled pattern) to minimise any rotational contribution. Since the snapping distances vary between elements due to the manufacturing deviation, the measured responses were adjusted for uniform comparison with those of numerical results. The details of the data adjustment can be found in Supplementary Information.

### Voltage Response

In this study, piezoelectric transducers (MIDE qp-16n) were used as the power conversion mechanisms to convert the induced strains into electrical currents. Two such transducers were glued near the roots (where the strains of the fundamental mode shape are the largest) of the bistable element with epoxy (Loctite E-120hp) and cured for 48 hours at room temperature (Supplementary Fig. 6). To obtain the voltage response, the piezoelectric elements were connected in parallel so that their electrical outputs combine constructively at the junction, and then a closed circuit was formed by connecting a resistor. The voltage difference across the resistor was measured through dSpace data acquisition system (DS1104) with 16-bit resolution. Various resistors with different resistance values and series connections of several resistors were used for the resistance sweep.

## Electronic supplementary material

Supplementary video 1 - Quasi-static input

Supplementary video 2 - Low intensity impulsive input

Supplementary video 3 - High intensity impulsive input

Supplementary video 4 - Breather mode

Supplementary Information
