# Supplementary material for: Input-Independent Energy Harvesting in Bistable Lattices from Transition Waves
Source: Sci Rep. 2018 Feb 26;8:3630. doi: 10.1038/s41598-018-22003-7 (PMC5827759; doi:10.1038/s41598-018-22003-7)
Supplement: Supplementary file 5 — Supplementary Information [file 41598_2018_22003_MOESM5_ESM.pdf]

SUPPLEMENTARY INFORMATION for:
Input-Independent Energy Harvesting in Bistable Lattices from Transition Waves

M. Hwang and A. F. Arrieta

January 17, 2018

1 Measured Force-Deflection Curves

Figure 1 shows the experimentally-fitted force-displacement curves for the bistable element with 0.225 m of rail distance and for magnetic interaction from the previous study, and the corresponding parameters are summarised in Tab. 1. Refer to the Supplemental Material of Ref. [33] in the manuscript for how these curves are obtained.

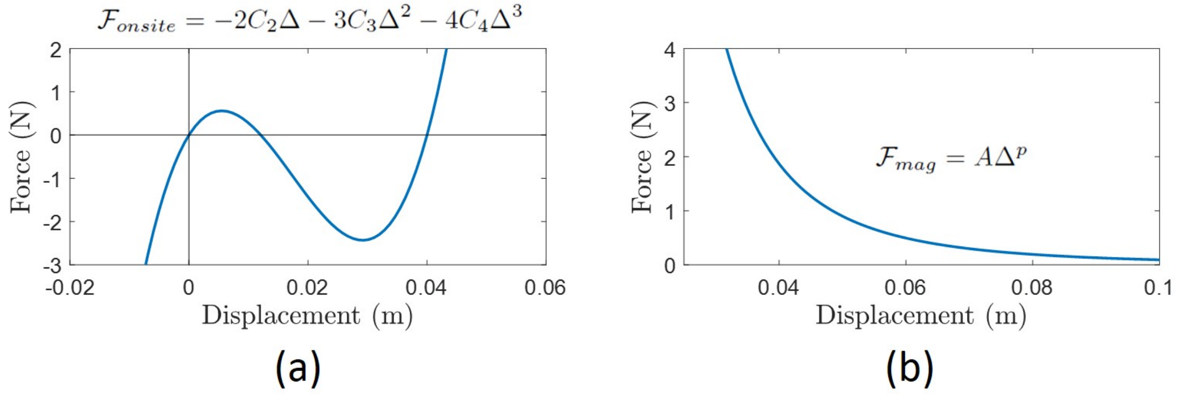

Figure 1: (a) Experimental force-deflection curves for the bistable element with the rail distance $R = 0.225$ m and (b) inter-element magnetic force.

A	p	C_2	C_3	C_4
4.95×10^{-5}	-3.274	217.101	-23,426	449,961

Table 1: System parameters for the baseline lattice

2 Phonon Transmission

Phonon transmission is obtained by linearising the governing equation about the stable equilibria, assuming a traveling wave solution of the form $\bar{u}_n = \bar{U}e^{i(\bar{k}n - \bar{\omega}\bar{t})}$, where $\bar{k}$ and $\bar{\omega}$ are the nondimensionalised wave number and frequency. The dispersion relation in the linear regime is obtained as

$$\bar{k} = \pm 2 \arcsin \sqrt{\frac{-\bar{\omega}^2 - i\bar{\omega}\bar{b} - \bar{\mathcal{F}}'_{onsite}(\bar{u}_n^*)}{4p}}, \tag{1}$$

where $\bar{u}_n^*$ represents a static equilibrium position, and the following nondimensionalisation is used:

$$\bar{k} = kL, \quad \bar{\omega} = \omega T, \quad \bar{b} = \frac{bT}{m}, \quad \bar{\mathcal{F}}_{onsite} = \frac{\mathcal{F}_{onsite}}{AL^p}, \quad T^2 = \frac{m}{AL^{p-1}}. \tag{2}$$

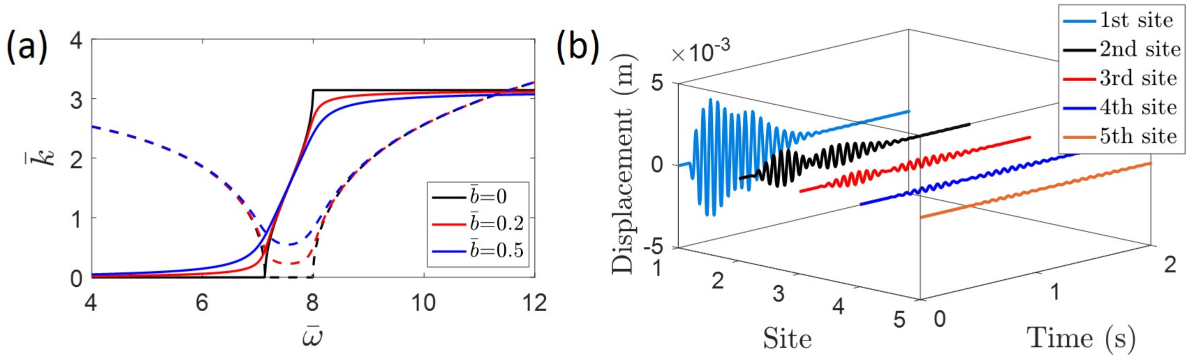

Figure 2: (a) Dispersion relation of the linearised equation: real (solid line) and imaginary (dashed line) parts of the nondimensionalised wave number. (b) Analytical solution of the phonon transmission in a lightly damped (b=0.2 Ns/m) lattice under sinusoidal input with a frequency (ω=105 rad/s) within the pass band.

In Fig. 2(a), this relation is plotted for different values of damping. For a conservative lattice ($\bar{b}$=0), the presence of the on-site potential acts as distributed elastic surroundings, resulting in a pass band between the low ($\bar{\omega}$=7.13) and high cutoff frequencies ($\bar{\omega}$=7.99). For dissipative systems ($\bar{b}$>0), $\bar{k}$ exhibits an imaginary part (dashed lines) which contributes to wave attenuation. Hence, phonons can never be stably transmitted in a physical setup. Figure 2(b) shows the responses of the first five elements in this small-amplitude regime of the lattice system with a low damping (b=0.2 Ns/m). The phonon transmission is almost completely disintegrated after passing through only a few elements.

3 Minimum Snapping Force in a Lattice

The force-deflection curve for a lattice of multiple unit cells is obtained numerically by applying a load quasi-statically to the first element and measuring the corresponding deflection at the same site. The minimum snapping force is defined as the smallest static force that triggers the state change. The numerical simulation shows the same force-deflection relation for the lattices with more than three unit cells.

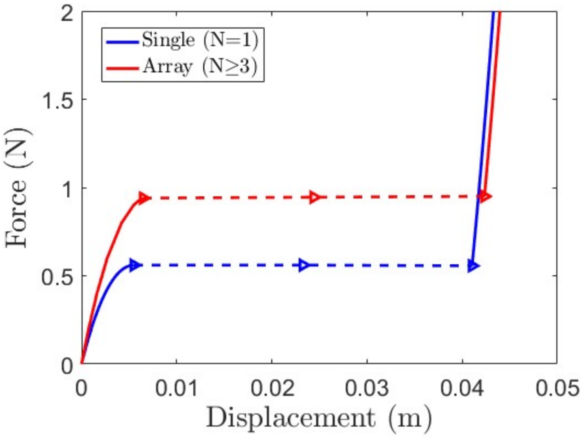

Figure 3: Minimum snapping force required to induce the state transition for a single bistable element and an array of bistable elements.

4 Experimental Responses

The experimental (dashed lines) and numerical responses (solid lines) for two selected cases (periodic lattice and $L_{defect}/L = 0.857$) are plotted in Figs. 4(a) and (b), respectively. To account for the manufacturing variability of the bistable elements, the experimental results are adjusted such that the snapping distances match those of numerical results. Although the details of the motion, such as the propagation velocity and the frequency of the free vibration after the completion of the state transition, do not match exactly, they still undergo qualitatively similar changes.

Possible reasons for the discrepancy are the limited order of the quartic potential approximation to describe the on-site dynamics produced by the bistable elements and the presence of coupled rotational motion. Nevertheless, the simplicity of the model used in this study captures the qualitative behaviour of the lattice, fulfilling the objectives of this investigation.

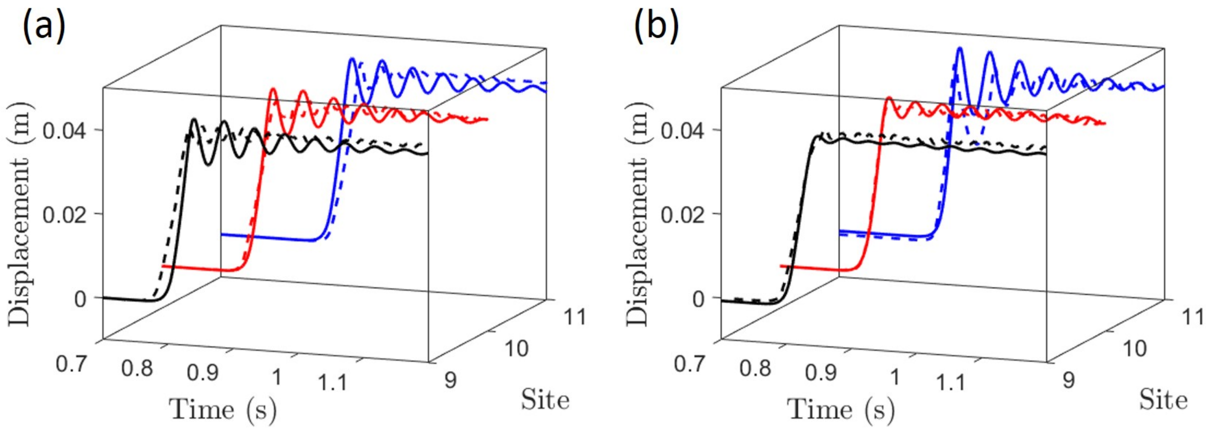

Figure 4: Numerical (solid lines) and experimental (dashed lines) responses around the control site for (a) the periodic lattice and (b) the lattice spacing defect ratio of 0.857.

5 Energy Analysis

Consider an enclosed system of an infinitely long lattice of bistable elements. At the instant t_0 a wave enters the system, the total available energy in the system is $E_{tot}|_{t_0} = E_{in} + N\Delta\phi$, where E_{in}, $\Delta\phi$, and N are the wave energy transmitted from outside the system, difference between two stable states of the bistable element, and the number of elements in the system. From the infiniteness of the selected section, $E_{in} \ll N\Delta\phi$ and thus $E_{tot}|_{t_0} \approx N\Delta\phi$. After all the wave train passed by (so that all the elements in the system reach the steady states at the lower equilibria), the total energy at this instant can be written as $E_{tot}|_{t_\infty} = Ne_{tot}|_{t_\infty}$ from periodicity of the lattice. e_{tot} is the total energy per unit element which can be expresses as $e_{tot} = ke + pe_{onsite} + pe_{mag} + de$, where ke, pe_{onsite}, pe_{mag}, and de are kinetic energy, on-site potential, potential due to magnetic interaction, and dissipated energy per unit element, respectively. $E_{tot}|_{t_0}$ should remain constant for the same set of bistable elements, and therefore $E_{tot}|_{t_\infty}$ also remain unaltered even if any other lattice parameters than the on-site potential change. In addition, e_{tot} should be conserved at any time instant from the conservation of total energy in an enclosed system.

Effect of Mass Change

From the above argument, e_{tot}'s of two different lattices with the same on-site elements are conserved:

$$ke^{(1)} + pe^{(1)}_{onsite} + pe^{(1)}_{mag} + de^{(1)} = ke^{(2)} + pe^{(2)}_{onsite} + pe^{(2)}_{mag} + de^{(2)}, \tag{3}$$

where superscript indicates a lattice with a different set of parameters. During the displacement of mass from zero to an arbitrary deflection Δ, it is reasonable to assume $|pe^{(2)}_{mag} - pe^{(1)}_{mag}| \ll 1$ from the compactness of the transition wave (can span as small as a single element according to Ref. [33]). This leads to

$$\begin{aligned} (\frac{1}{2}mv^2)^{(1)} + \cancel{pe_{onsite}|_{x=\Delta}} + (\int_{t(x=0)}^{t(x=\Delta)} bv^2 dt)^{(1)} &= (\frac{1}{2}mv^2)^{(2)} + \cancel{pe_{onsite}|_{x=\Delta}} + (\int_{t(x=0)}^{t(x=\Delta)} bv^2 dt)^{(2)} \\ \Rightarrow (\frac{1}{2}mv^2)^{(1)} + (\int_0^{\Delta} bvdx)^{(1)} &= (\frac{1}{2}mv^2)^{(2)} + (\int_0^{\Delta} bvdx)^{(2)}. \end{aligned} \tag{4}$$

Letting $m^{(1)} > m^{(2)}$ and assuming a fixed b, it can be easily concluded that $v^{(1)} < v^{(2)}$ for all Δ. Now, dissecting the energy components when the element reaches its maximum potential (therefore $ke = 0$),

$$pe_{onsite}|_{x=\Delta_1} + \int_0^{\Delta_1} bv^{(1)} dx = pe_{onsite}|_{x=\Delta_2} + \int_0^{\Delta_2} bv^{(2)} dx. \tag{5}$$

If $\Delta_1 < \Delta_2$, then $pe_{onsite}|_{x=\Delta_1} < pe_{onsite}|_{x=\Delta_2}$. The equality (5) can never hold since $v^{(1)}$ is always less than $v^{(2)}$ ($\int_0^{\Delta_1} bv^{(1)}dx < \int_0^{\Delta_2} bv^{(2)}dx$). On the other hand, the equality can satisfy for $\Delta_1 > \Delta_2$, which conclude that the a larger mass yields a larger amplitude.

Effect of Inter-element Forcing Change

As derived in the manuscript, $pe_{mag}^{(1,2)} = -\frac{A}{p+1}(x_2 - x_1 + L^{(1,2)})^{p+1}$. Since $p+1$ is in general negative, $pe_{mag}^{(1)} < pe_{mag}^{(2)}$ for $L^{(1)} > L^{(2)}$. Substituting this relation into Eq. (3), we obtain the following inequality:

$$(\frac{1}{2}mv^2)^{(1)} + \cancel{pe_{onsite}|_{x=\Delta}} + (\int_0^{\Delta} bvdx)^{(1)} < (\frac{1}{2}mv^2)^{(2)} + \cancel{pe_{onsite}|_{x=\Delta}} + (\int_0^{\Delta} bvdx)^{(2)}. \quad (6)$$

Similar to the case of mass change, it can be shown that $v^{(1)} < v^{(2)}$ for all Δ if $L^{(1)} > L^{(2)}$. At the instant that pe becomes maximum, the inequality becomes

$$pe_{onsite}|_{x=\Delta_1} + \int_0^{\Delta_1} bv^{(1)}dx < pe_{onsite}|_{x=\Delta_2} + \int_0^{\Delta_2} bv^{(2)}dx. \quad (7)$$

Following the same process, if $\Delta_1 > \Delta_2$, $pe_{onsite}|_{x=\Delta_1} > pe_{onsite}|_{x=\Delta_2}$. The inequality may be satisfied this time since $v^{(1)} < v^{(2)}$. If $\Delta_1 < \Delta_2$, then $pe_{onsite}|_{x=\Delta_1} < pe_{onsite}|_{x=\Delta_2}$ and $\int_0^{\Delta_1} bv^{(1)}dx < \int_0^{\Delta_2} bv^{(2)}dx$ so that the inequality always holds. Therefore, the effect of the inter-element forcing change (or lattice spacing change) is inconclusive; it may or may not induce a larger amplitude.

6 Time Progression of the Breather-like Mode

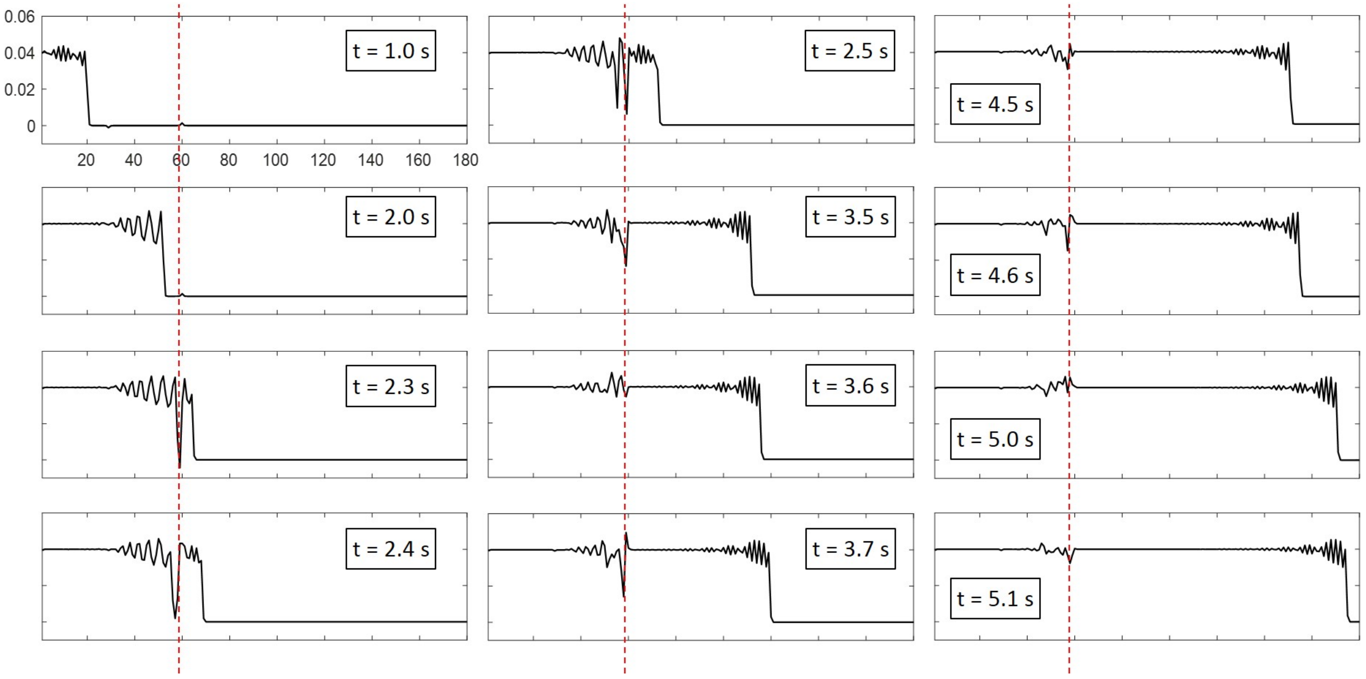

Figure 5: Time progression of a breather-like mode in the spatial configuration. A spatially localised inter-well oscillation is generated while the main transition wave continues to propagate. The dashed red line indicates the boundary (59th site) of the defect section.

7 Experimental Setup for Electromechanical Conversion Performance

Figure 6(a) shows the bistable element with embedded piezoelectric transducers and its placement in the experimental setup. The specific location of the transducers are determined based on the FE analysis [Fig. 6(b)], which shows that the largest in-plain strains of the fundamental mode in the length direction of the element occur near the roots.

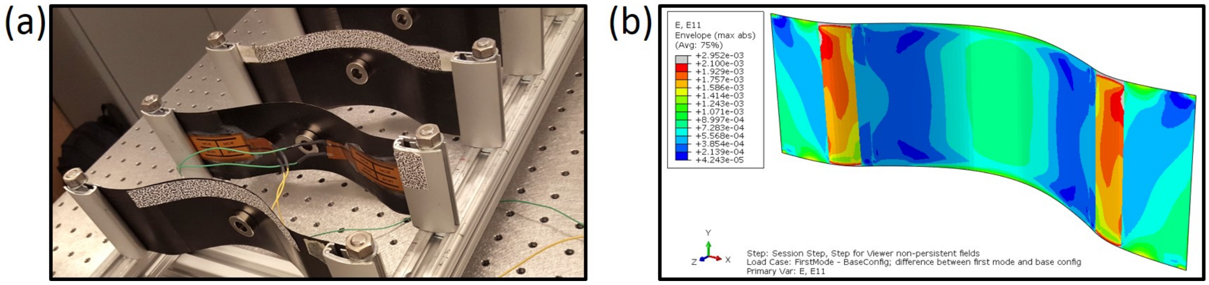

Figure 6: (a) Piezoelectric transducer attachment to a bistable element and its installation within the lattice. (b) In-plane strain distribution of the fundamental mode in the length direction of the bistable element from FE analysis.
